# Supplementary material for: In-line digital holographic microscopy using a consumer scanner
Source: Sci Rep. 2013 Sep 16;3:2664. doi: 10.1038/srep02664 (PMC3773619; doi:10.1038/srep02664)
Supplement: Supplementary Information [file srep02664-s2.pdf]

Supplementary Information

Article Title:
In-line digital holographic microscopy using a consumer scanner

Authors：
Tomoyoshi Shimobaba, Hiroya Yamanashi, Takashi Kakue, Minoru Oikawa, Naohisa Okada, Yutaka Endo, Ryuji Hirayama, Nobuyuki Masuda, Tomoyoshi Ito

File name of the movie：movie.mov

The movie is associated with Figure 2.
